# Frequency of Early Microvascular Changes in Prediabetic Individuals: A Focus on Renal, Retinal, and Peripheral Nerve Involvement

**DOI:** 10.7759/cureus.92040

**Published:** 2025-09-11

**Authors:** Ayesha Rauf, Hafiz Zunair Iqbal, Muhammad Zohaib, Mahnoor Naeem, Tayyaba Arooj Mufti, Humza Sadique, Adeel Ahmed, Muhammad Irfan Jamil, Muhammad Ayoob Memon, FNU Anam

**Affiliations:** 1 Acute/Internal Medicine, Lahore General Hospital, Lahore, PAK; 2 Internal Medicine, Akhtar Saeed Medical and Dental College, Lahore, PAK; 3 Acute Care, NES Healthcare UK, Edinburgh, GBR; 4 Acute Care, Pakistan Kidney and Liver Institute and Research Center, Lahore, PAK; 5 Medicine, King Edward Medical University, Lahore, PAK; 6 Cardiology/Medicine, Akhtar Saeed Medical and Dental College, Lahore, PAK; 7 Medicine, South Tyneside and Sunderland NHS Foundation Trust, Sunderland, GBR; 8 Medicine, Lahore General Hospital, Lahore, PAK; 9 Anesthesiology and Critical Care, King Edward Medical University, Lahore, PAK; 10 Nephrology, Lahore General Hospital, Lahore, PAK; 11 Internal Medicine, Jinnah Sindh Medical University, Karachi, PAK; 12 Medicine, Sayed Abdullah Shah Institute of Medical Sciences, Sehwan, PAK

**Keywords:** diabetic microvascular complications, diabetic peripheral neuropathy (dpn), microalbuminuria, prediabetes, retinopathy

## Abstract

Background and objective: Prediabetes is increasingly recognized as a stage where microvascular injury begins, particularly involving renal, retinal, and neural pathways. Early detection of such changes may guide timely interventions. This study aimed to determine the frequency of early microvascular complications in prediabetic individuals and evaluate their association with clinical and biochemical risk factors.

Methods: This cross-sectional observational study was conducted at Lahore General Hospital, Lahore, Pakistan, from July 2023 to June 2024. A total of 268 prediabetic adults, defined by the American Diabetes Association (ADA) 2023 criteria, were enrolled using non-probability consecutive sampling. Microvascular outcomes were assessed by urine albumin-to-creatinine ratio (UACR), the Michigan Neuropathy Screening Instrument, and the Early Treatment Diabetic Retinopathy Study (ETDRS)-based fundus photography. Biochemical tests included fasting glucose, two-hour oral glucose tolerance test (OGTT), and glycated hemoglobin (HbA1c).

Results: In this study of 268 prediabetic individuals, the mean age was 46.07±7.65 years, with a higher mean age among those with microvascular changes (48.44±9.21 vs. 45.64±7.28 years; p=0.031). The mean body mass index (BMI) was 27.99±3.59 kg/m², with significantly higher values in those with microvascular changes (p<0.001). Microvascular changes were most frequent in overweight (48.8%) and obese (36.6%) participants compared to those of normal weight (14.6%; p=0.017). Hypertension (58.5% vs. 39.6%; p=0.024) and dyslipidemia (78% vs. 51.5%; p=0.002) were significantly associated, whereas smoking (p=0.126) and family history of diabetes (p=0.296) were not. Biochemical parameters were significantly higher in those with microvascular changes: fasting plasma glucose (p<0.001), two-hour OGTT (p<0.001), HbA1c (6.11% vs. 5.92%; p=0.001), and UACR (p<0.001). Microalbuminuria was detected in 10.1%, retinopathy in 4.1%, and peripheral neuropathy in 7.8%.

Conclusion: Early microvascular changes were observed in prediabetes, with significant associations with age, BMI, hypertension, dyslipidemia, and glycemic indices. Screening for albuminuria, retinopathy, and neuropathy may enable timely interventions to prevent progression to diabetes and related vascular complications.

## Introduction

Prediabetes, defined by the presence of impaired fasting glucose, impaired glucose tolerance, or glycated hemoglobin (HbA1c) levels between normal and diabetic thresholds, is a well-recognized intermediate stage in the natural history of type 2 diabetes mellitus [1]. It affects 374 million people globally (7.3%) and is projected to rise to 8.3% by 2045 [2]. In Pakistan, its prevalence is estimated at 14.4%, underscoring the urgency of early detection [3]. The public health implications are considerable, as prediabetes not only predicts progression to overt diabetes but is also linked with increased cardiovascular morbidity and mortality [4]. South Asia carries a disproportionate share of this burden due to a combination of genetic susceptibility, lifestyle factors, and limited access to early preventive interventions [5,6].

Microvascular injury can develop in the prediabetic stage, preceding overt diabetes. Mild, chronic hyperglycemia and postprandial glucose fluctuations trigger oxidative stress, low-grade inflammation, endothelial dysfunction, and activation of the polyol and protein kinase C pathways, leading to reduced nitric oxide, increased vascular permeability, and basement membrane thickening [7,8]. Renal involvement may present as early hyperfiltration and microalbuminuria in prediabetic individuals [9]. Retinal changes include capillary non-perfusion, pericyte loss, and early blood-retinal barrier breakdown, and peripheral nerve injury arises from microvascular insufficiency and metabolic damage to Schwann cells and axons. These subclinical alterations underscore the concept that prediabetes is not a metabolically benign state but one in which early tissue injury is already underway across multiple organ systems [10,11]. Recent evidence indicates that microvascular changes are present in the prediabetic stage, though prevalence rates vary. Shrestha et al. found diabetic retinopathy in 5.67% of 141 prediabetic individuals aged 40-79 years, all with mild non-proliferative changes [12]. In a study of 2,713 individuals, prediabetes prevalence was 28% with isolated microalbuminuria in 6.5% [13]. Another study of 8,775 participants identified prediabetes in 45% with higher median urine albumin-to-creatinine ratio (UACR) (3.5 mg/g vs. 2.6 mg/g) and greater microalbuminuria prevalence (6.3% vs. 3.6%; p<0.001) compared with normal glucose regulation [14]. Butler et al., in a systematic review and meta-analysis, reported a pooled prevalence of 4% for retinopathy, 10.5% for nephropathy, and 2.5% for neuropathy [15]. The findings suggest that while higher HbA1c is linked to greater risk, nephropathy and other microvascular complications can occur below the diagnostic threshold.

Early microvascular changes in prediabetes are recognized but show wide variability in reported prevalence. Existing studies often assess single complications and rarely evaluate renal, retinal, and peripheral nerve involvement together [16]. The present study was undertaken to determine the frequency of early renal (albuminuria), retinal, and peripheral nerve involvement in individuals with prediabetes.

## Materials and methods

This cross-sectional observational study was executed at Lahore General Hospital, Lahore, Pakistan, from July 2023 to June 2024, following authorization from the institute's Institutional Review Board (approval number: 110/07/2023). A non-probability consecutive sampling was used to enroll patients. A sample size of 268 was calculated based on a hypothesized prevalence of microalbuminuria of 12.8%, with an absolute precision of 4% and a 95% confidence level [17].

Adults aged 18-65 years of either gender classified as prediabetic according to the American Diabetes Association (ADA) 2023 criteria [18], defined by fasting plasma glucose between 100 and 125 mg/dL, two-hour oral glucose tolerance test (OGTT) glucose between 140 and 199 mg/dL, or HbA1c between 5.7% and 6.4%, and who provided written informed consent were included. Patients were excluded if they had a diagnosis of type 1 or type 2 diabetes mellitus, macrovascular disease such as ischemic heart disease, stroke, or peripheral arterial disease, chronic kidney disease with an estimated glomerular filtration rate (eGFR) of less than 60 mL/min/1.73 m², or urinary tract infection. Retinal disorders unrelated to glycemic status, neurological conditions from other etiologies (e.g., stroke, B12 deficiency, spinal pathology), pregnancy, or systemic inflammatory disease were also excluded.

Written informed consent was obtained from each participant prior to enrollment. Demographic and clinical data, including age, sex, body mass index (BMI), smoking status, family history of diabetes, hypertension, and dyslipidemia, were recorded. Laboratory investigations were performed following an overnight fast of 8-12 hours. Fasting plasma glucose and two-hour post-load glucose were analyzed using the glucose oxidase-peroxidase enzymatic method. HbA1c was measured via high-performance liquid chromatography (HPLC). Assessment of nephropathy was carried out using the UACR, measured from the second early morning midstream urine sample collected in sterile containers. The analysis was performed using a standardized turbidimetric immunoassay technique, which is a validated method for quantifying microalbuminuria. Participants with UACR ≥30 mg/g were classified as having nephropathy. 

Peripheral neuropathy was assessed using the physical examination component of the Michigan Neuropathy Screening Instrument (MNSI), a validated and widely used clinical tool. The examination included inspection of both feet for deformities, calluses, infections, and dryness; testing of vibration perception using a 128-Hz tuning fork applied to the dorsal hallux; assessment of ankle reflexes using a percussion hammer; and monofilament testing at four standard plantar sites on each foot using a 10-gram Semmes-Weinstein monofilament. A total score of ≥2.5 on the MNSI physical examination was considered diagnostic of peripheral neuropathy [19].

Retinal assessment was performed using seven-field stereoscopic digital fundus photography according to the protocol established by the Early Treatment Diabetic Retinopathy Study (ETDRS) [20]. Participants underwent pharmacological pupil dilation with tropicamide 1% and phenylephrine 2.5% eye drops, after which high-resolution fundus photographs were obtained for both eyes using a non-contact digital fundus camera. Retinopathy was classified based on the ETDRS criteria, and only non-proliferative diabetic retinopathy (NPDR) was considered for this study, in keeping with the prediabetic status of the study population. All assessments were conducted by trained clinicians who were blinded to the participants' biochemical results in order to minimize observer bias.

Data were entered and analyzed using IBM SPSS Statistics for Windows, Version 26.0 (Released 2019; IBM Corp., Armonk, New York, United States). Continuous variables were expressed as mean±standard deviation (SD). Categorical variables were presented as frequencies and percentages. The association between categorical variables was assessed using the chi-squared (χ²) test, and odds ratios (OR) with 95% confidence intervals (CI) were calculated where appropriate. For continuous variables, comparisons between patients with and without microvascular changes were performed using the independent samples t-test. Mean differences (MD) with 95% CI were reported. A p-value of less than 0.05 was considered statistically significant.

## Results

In this study of 268 participants, the mean age was 46.07±7.65 years, with a significantly higher age observed in those with microvascular changes (48.44±9.21 vs. 45.64±7.28 years; t=2.167; p=0.031). Males comprised 47% of the cohort, but gender was not significantly associated with microvascular changes (χ²=2.579; p=0.108). Mean BMI was 27.99±3.59 kg/m² and was significantly higher in the microvascular change group (30.48±4.29 vs. 27.54±3.27; t=5.026; p<0.001). BMI category analysis showed a significant association (χ²=8.194; p=0.017), with microvascular changes most frequent in the overweight group (48.8%), followed by obese participants (36.6%) and normal-weight participants (14.6%). Smoking status was not significantly associated (χ²=4.151; p=0.126). A family history of diabetes showed no significant relationship (χ²=1.093; p=0.296). Significant associations were observed for hypertension (χ²=5.069; p=0.024) and dyslipidemia (χ²=9.884; p=0.002) with microvascular changes (Table 1).

**Table 1:** Baseline characteristics and association of clinical variables with microvascular changes Continuous variables are presented as mean±SD and were analyzed by the independent samples t-test. Categorical variables are presented as number and percentage and were analyzed by Pearson's chi-squared (χ²) test. The effect size is reported as the mean difference for numerical variables and OR with 95% CI for categorical variables. BMI: body mass index; OR: odds ratio; MD: mean difference; CI: confidence interval

Variable	Category	Total (n=268)	With microvascular changes (n=41)	Without microvascular changes (n=227)	Test statistic (χ²/t-value)	Effect size (OR/MD) (95% CI)	P-value
Age (years)	-	46.07±7.65	48.44±9.21	45.64±7.28	t=2.167	MD=2.79 (0.25-5.34)	0.031
Gender	Male	126 (47%)	24 (58.5%)	102 (44.9%)	χ²=2.579	OR=1.73 (0.88-3.39)	0.108
	Female	142 (53%)	17 (41.5%)	125 (55.1%)			
BMI (kg/m²)	-	27.99±3.59	30.48±4.29	27.54±3.27	t=5.026	MD=2.93 (1.78-4.08)	<0.001
BMI category (kg/m²)	Normal (18.5-24.9)	89 (33.2%)	6 (14.6%)	83 (36.6%)	χ²=8.194	-	0.017
	Overweight (25-29.9)	112 (41.8%)	20 (48.8%)	92 (40.5%)			
	Obese (≥30)	67 (25%)	15 (36.6%)	52 (22.9%)			
Smoking status	Non-smoker	162 (60.4%)	21 (51.2%)	141 (62.1%)	χ²=4.151	-	0.126
	Ex-smoker	47 (17.5%)	6 (14.6%)	41 (18.1%)			
	Current-smoker	59 (22%)	14 (34.1%)	45 (19.8%)			
Family history of diabetes	Yes	176 (65.7%)	24 (58.5%)	152 (67%)	χ²=1.093	OR=0.69 (0.35-1.37)	0.296
Hypertension	Yes	114 (42.5%)	24 (58.5%)	90 (39.6%)	χ²=5.069	OR=2.14 (1.09-4.22)	0.024
Dyslipidemia	Yes	149 (55.6%)	32 (78%)	117 (51.5%)	χ²=9.884	OR=3.34 (1.52-7.32)	0.002

In 268 participants, the mean fasting plasma glucose was 109.07±6.61 mg/dL, higher in those with microvascular changes (114.16±6.73 vs. 108.16±6.17; t=5.653; p<0.001). The mean two-hour OGTT glucose was 154.81±8.80 mg/dL, elevated in the affected group (161.29±13.11 vs. 153.64±7.21; t=5.388; p<0.001). HbA1c was 5.95±0.23%, higher in those with microvascular changes (6.11±0.25 vs. 5.92±0.21; t=5.128; p=0.001). UACR averaged 24.10±7.97 mg/g, markedly higher in affected patients (36.25±11.49 vs. 21.90±4.48; t=13.916; p<0.001) (Table 2).

**Table 2:** Comparison of biochemical parameters between patients with and without microvascular changes Data are expressed as mean±standard deviation (SD). Statistical analysis was performed using the independent samples t-test. OGTT: oral glucose tolerance test; HbA1c: glycated hemoglobin; CI: confidence interval

Variable	Total (n=268)	With microvascular changes (n=41)	Without microvascular changes (n=227)	t-value	Mean difference (95% CI)	P-value
Fasting plasma glucose (mg/dL)	109.07±6.61	114.16±6.73	108.16±6.17	5.653	5.99 (3.90-8.09)	<0.001
Two-hour OGTT glucose (mg/dL)	154.81±8.80	161.29±13.11	153.64±7.21	5.388	7.65 (4.85-10.44)	<0.001
HbA1c (%)	5.95±0.23	6.11±0.25	5.92±0.21	5.128	0.18 (0.12-0.26)	0.001
Urine albumin-to-creatinine ratio (mg/g)	24.10±7.97	36.25±11.49	21.90±4.48	13.916	14.34 (12.32-16.38)	<0.001

**Figure 1:**
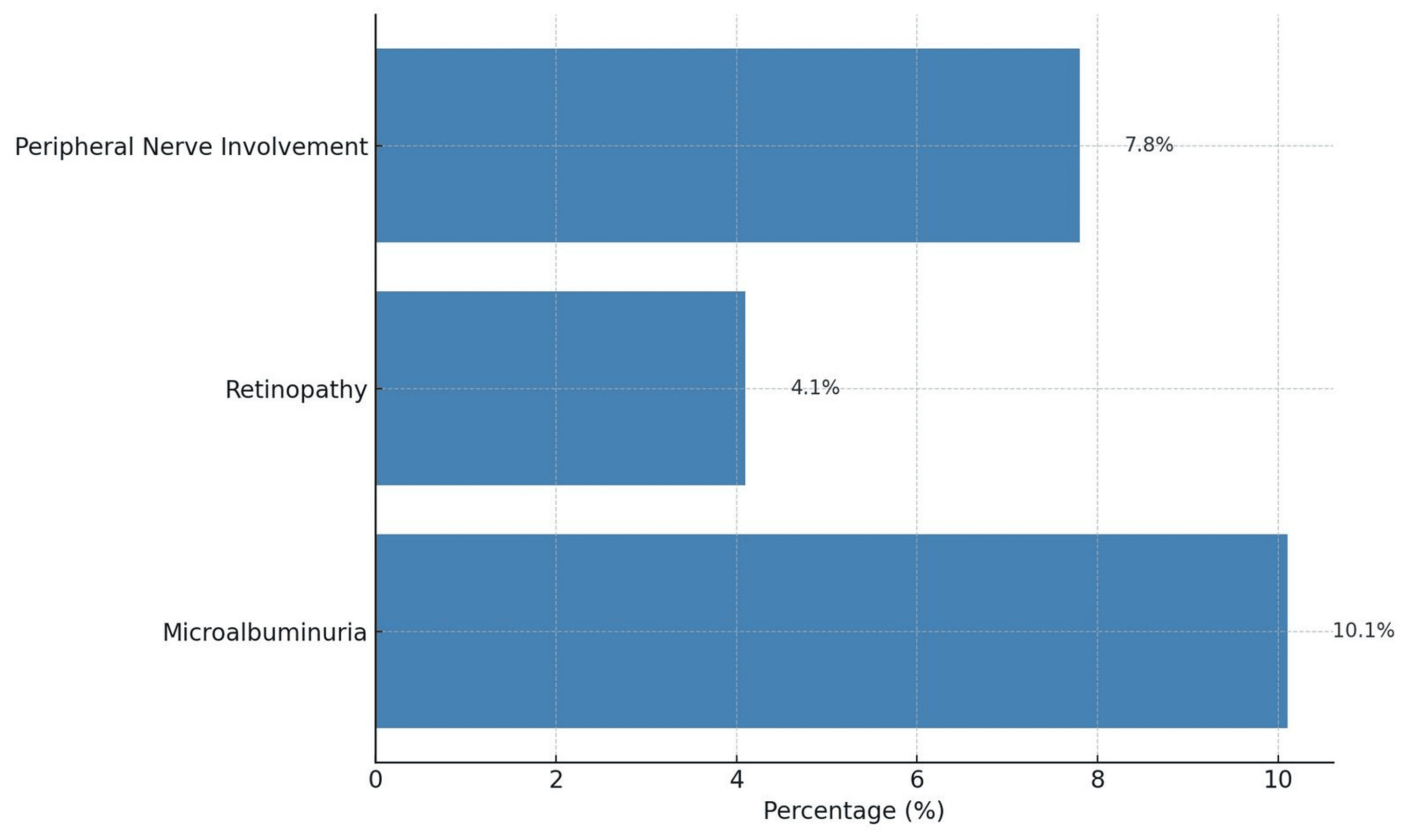
Frequency of early microvascular changes

In the present study, microvascular complications were observed with varying frequencies. Microalbuminuria was present in 27 (10.1%) participants, retinopathy in 11 (4.1%), and peripheral nerve involvement in 21 (7.8%) (Figure 1).

## Discussion

The present study investigated the frequency of early microvascular changes among prediabetic individuals, focusing on albuminuria, retinal abnormalities, and peripheral nerve involvement. Among 268 participants, microalbuminuria was detected in 27 (10.1%), retinopathy in 11 (4.1%), and peripheral nerve involvement in 21 (7.8%). These findings confirm that microvascular complications are not confined to overt diabetes but emerge during the prediabetic stage, reinforcing the need for early risk stratification and preventive strategies. The mean age of study participants was 46.07±7.65 years, with a significantly higher age among those with microvascular changes (48.44±9.21 vs. 45.64±7.28 years; p=0.031). Older age has been consistently identified as a determinant of microvascular involvement in prediabetes. The large-scale meta-analysis by Sune et al., encompassing 10,539 prediabetic individuals across 26 countries, highlighted age >50 years as an independent predictor of retinopathy prevalence, with risk varying across ethnicities [21]. Similarly, Mairaj et al. demonstrated that prediabetic individuals with neuropathy had significantly higher mean age (55.6 vs. 45.9 years; p<0.001) [22]. Together with our findings, these data suggest that advancing age, even within the prediabetic window, contributes substantially to the initiation of microvascular injury.

BMI emerged as a strong predictor of microvascular involvement in our population. Participants with microvascular changes had a significantly higher BMI (30.48±4.29 vs. 27.54±3.27 kg/m²; p<0.001). Stratified analysis revealed that overweight (48.8%) and obese (36.6%) individuals were more likely to manifest complications compared to those with normal BMI (14.6%; p=0.017). This aligns with observations by Mairaj et al., who reported higher BMI among prediabetics with neuropathy (25.6 vs. 23.1 kg/m²; p<0.001), and Körei et al., where high-risk prediabetics (Finnish Diabetes Risk Score (FINDRISC) ≥12) had significantly higher BMI (29.9 vs. 25.9 kg/m²; p<0.001) and greater neuropathy burden [22,23]. Collectively, these findings underscore the central role of obesity as a modifiable determinant of early microvascular injury.

Traditional risk factors such as hypertension and dyslipidemia also demonstrated significant associations. In our cohort, microvascular changes were more frequent among hypertensive participants (58.5% vs. 39.6%; p=0.024) and those with dyslipidemia (78% vs. 51.5%; p=0.002). This observation is concordant with prior studies: Palladino et al. reported dyslipidemia in 63.3% of prediabetics with microvascular disease, and Sune et al. noted hypertension in up to 71% of prediabetic participants with retinopathy [21,24]. These findings reinforce the synergistic effect of metabolic and hemodynamic risk factors in accelerating microvascular pathology before the onset of diabetes.

Our study demonstrated that prediabetic individuals with microvascular changes had significantly higher fasting plasma glucose (114.16±6.73 vs. 108.16±6.17 mg/dL; p<0.001), two-hour OGTT glucose (161.29±13.11 vs. 153.64±7.21 mg/dL; p<0.001), and HbA1c levels (6.11±0.25% vs. 5.92±0.21%; p=0.001). Elevated UACR was also strongly associated (p<0.001). Comparable findings were reported in prior literature. Perreault et al., using the longitudinal Diabetes Prevention Program Outcomes Study (DPPOS) data, showed that lower HbA1c and regression to normoglycemia were associated with reduced microvascular outcomes, though this effect was attenuated after adjusting for long-term glycemic exposure [25]. Butler et al. demonstrated that microvascular complications occur even at HbA1c <6.5%, with a retinopathy prevalence of 2.4% in the 6-6.4% group, confirming that risk is not confined to diagnostic diabetes thresholds [15]. Mas-Fontao et al. in the Early Prevention of Diabetes Complications in People with Hyperglycemia (ePREDICE) trial similarly observed albuminuria in 4.65% of prediabetics, particularly in those with higher post-load glucose levels [26]. Together, these findings confirm that early vascular injury is closely linked to subtle but chronic glycemic elevations.

Microalbuminuria was observed in 10.1% of our participants, closely aligning with the prevalence reported by Sune et al. (11%) in their meta-analysis of prediabetic populations [21]. Venkatesh et al. reported a prevalence of 12.8%, with higher occurrence in individuals with combined impaired fasting glucose and impaired glucose tolerance [17]. Palladino et al. documented a nephropathy prevalence of 23.8% among prediabetic individuals, with adjusted odds ratios significantly elevated compared to normoglycemia [24]. In contrast, Kalra and Kour observed nephropathy in only 2% of prediabetic patients [27]. The variability across studies may reflect differences in diagnostic criteria, population risk profiles, and duration of glycemic exposure. The early detection of albuminuria in prediabetes is therefore clinically relevant as a marker of generalized endothelial dysfunction and a predictor of progression to overt diabetes.

Retinopathy was identified in 4.1% of participants in the present study, which falls within the range reported by previous meta-analyses and observational studies. Sune et al. reported a pooled prevalence of 8.1% (IQR: 4.2-11%), with higher estimates in studies using the World Health Organization (WHO) criteria. Butler et al. found a retinopathy prevalence of 2.4% in the HbA1c 6-6.4% category and 7.97% in those ≥6.5% [21]. Palladino et al. documented a higher prevalence of retinopathy among prediabetic patients (25.2%) compared to normoglycemic individuals (13.9%), with an adjusted odds ratio of 1.76 (95% CI: 1.69-1.85) [24]. However, other studies, such as Li Rudvan et al. and Venkatesh et al., reported no detectable retinopathic changes in prediabetic participants, highlighting methodological variability [17,28]. Our observed prevalence supports the existence of early retinal damage in prediabetes, though at a lower frequency compared to nephropathy.

Peripheral nerve involvement was documented in 7.8% of our participants. Mairaj et al. reported a neuropathy prevalence of 12.3% in prediabetes, associated with higher BMI, cholesterol, and glycemic parameters, alongside universal hypertension and albuminuria in affected individuals [22]. Körei et al. found a higher prevalence of neuropathy symptoms and objective sensory deficits among high-risk prediabetics, with HbA1c independently predicting parasympathetic dysfunction [23]. Butler et al. reported a lower pooled prevalence (2.47%), though with high heterogeneity across included studies [15]. Our findings add to the evidence that neuropathy manifests early in prediabetes, albeit with modest frequency compared to albuminuria. 

The strengths of this study include its adequate sample size, standardized assessment of biochemical and clinical parameters, and evaluation of multiple microvascular outcomes (albuminuria, retinopathy, neuropathy) in prediabetic individuals. However, its cross-sectional design limits causal inference, and reliance on single-center recruitment may restrict generalizability. The absence of advanced imaging modalities, such as optical coherence tomography angiography, and nerve conduction studies may have underestimated subtle changes. The absence of a healthy control group precluded direct comparison with normoglycemic individuals, and the use of non-probability consecutive sampling may introduce selection bias. Future studies should employ longitudinal, multicenter designs with comprehensive microvascular assessments to establish temporal associations, validate early biomarkers, and evaluate the impact of targeted interventions on microvascular complications in prediabetes.

## Conclusions

This study establishes that early microvascular changes are evident among individuals with prediabetes, indicating that vascular injury begins well before the onset of overt diabetes. Factors such as excess body weight, hypertension, dyslipidemia, and poor glycemic control were significantly related to these early complications, whereas sex, smoking, and family history showed no clear association. These findings emphasize the need for early risk stratification and targeted preventive measures. Incorporating screening for renal, retinal, and neurological changes into prediabetes management may help identify high-risk individuals at an earlier stage, thereby enabling timely interventions to reduce future diabetic complications.
